# The Role of the ^18^F-FDG PET/CT in the Management of Patients Suspected of Cardiac Implantable Electronic Devices’ Infection

**DOI:** 10.3390/jpm14010065

**Published:** 2024-01-04

**Authors:** Antonio Rosario Pisani, Dino Rubini, Corinna Altini, Rossella Ruta, Maria Gazzilli, Angela Sardaro, Francesca Iuele, Nicola Maggialetti, Giuseppe Rubini

**Affiliations:** 1 Interdisciplinary Department of Medicine, Section of Nuclear Medicine, University of Bari “Aldo Moro”, Policlinic of Bari, Piazza Giulio Cesare 11, 70124 Bari, Italy; corinna.altini@policlinico.ba.it (C.A.); rossella.ruta@yahoo.it (R.R.); francesca.iuele@policlinico.ba.it (F.I.); giuseppe.rubini@uniba.it (G.R.); 2 Radiotherapy, Precision Medicine Department, University of Campania “Luigi Vanvitelli”, 80138 Naples, Italy; rubini.dino@libero.it; 3 Nuclear Medicine, ASL Bari—Di Venere, 70131 Bari, Italy; marinagazzilli@msn.com; 4 Interdisciplinary Department of Medicine, Section of Radiology and Radiation Oncology, University of Bari “Aldo Moro”, Policlinic of Bari, Piazza Giulio Cesare 11, 70124 Bari, Italy; angela.sardaro@uniba.it (A.S.); nicola.maggialetti@uniba.it (N.M.)

**Keywords:** 18F-FDG PET/CT, nuclear cardiology imaging, cardiac implantable electronic devices, hybrid imaging

## Abstract

**Background:** Infection of Cardiac Implantable Electronic Devices (CIEDI) is a real public health problem. The main aim of this study was to determine the diagnostic performance of ^18^F-FDG PET/CT in the diagnosis of CIEDI. **Methods:** A total of 48 patients, who performed ^18^F-FDG PET/CT for the clinical suspicion of CIEDI were retrospectively analyzed; all patients were provided with a model with procedural recommendations before the exam. Sensitivity (Se), specificity (Sp), positive predictive value (PPV), negative predictive value (NPV) and diagnostic accuracy (DA) of ^18^F-FDG PET/CT were calculated; the reproducibility of qualitative analysis was assessed with Cohen’s κ test. The semi-quantitative parameters (SUVmax, SQR and TBR) were evaluated in CIEDI+ and CIEDI− patients using the Student’ *t*-test; ROC curves were elaborated to detect cut-off values. The trend of image quality with regards to procedural recommendation adherence was evaluated. **Results:** Se, Sp, PPV, NPV and DA were respectively 96.2%, 81.8%, 86.2%, 94.7% and 89.6%. The reproducibility of qualitative analysis was excellent (K = 0.89). Semiquantitative parameters resulted statistically different in CIEDI+ and CIEDI− patients. Cut-off values were SUV_max_ = 2.625, SQR = 3.766 and TBR = 1.29. Trend curves showed increasing image quality due to adherence to procedural recommendations. **Conclusions:** ^18^F-FDG-PET/CT is a valid tool in the management of patients suspected of CIEDI and adherence to procedural recommendations improves its image quality.

## 1. Introduction

In recent decades, scientific-technological development has led to a great improvement in Cardiac Implantable Electronic Devices (CIEDs) [1,2,3]. At the same time, the epidemiological profile of the population has changed, with a lengthening of the average lifetime, a parallel increase in the number of patients suffering from numerous comorbidities or immune-compromised diseses, and a greater incidence of age-related cardiovascular events [1,2,3]. Therefore, the indication for CIEDs’ implantation has significantly increased, as they are fundamental devices for numerous cardiac diseases that can compromise patients’ lives [4,5,6].

One of the more severe problems associated with the use of CIEDs is the infection of the device (Cardiac Implantable Electronic Device Infection, CIEDI). Despite preventive measures, the growing number of CIEDs implanted is associated with a disproportionate increase in the incidence of CIEDIs [7,8,9]. This complication is a real public health problem as it is associated with a severe prognosis and is related to significant morbidity, mortality and public health costs.

The clinical presentation of these infections is often misleading as it can be extremely variable, with a spectrum of manifestations ranging from nonspecific fever or simple superficial skin infection to the development of endocarditis, leading to a delay in clinical recognition [10,11,12,13].

Thus, prompt diagnosis is the crucial turning issue in conducting patients with CIEDI to guarantee them the best single-tailored diagnostic-therapeutic approach. Even if echocardiography is still the most used and reference imaging technique for CIEDI diagnosis, the use of multimodality imaging techniques is often strategic for optimal management of this complex clinical condition, as recently recommended in the 2020 International Consensus Document of the European Heart Rhythm Association, approved by many expert societies, and in the even more recently updated 2023 ESC Guidelines for the management of endocarditis [14,15,16,17].

In this context, 18F-fluorodeoxyglucose positron emission tomography/computed tomography (^18^F-FDG PET/CT) has a fundamental and growing role. In addition to its proven validity in the oncology field, this nuclear medicine technique is assuming an increasingly relevant position in the study of numerous inflammatory and infectious diseases, including cardiovascular ones [18,19,20,21,22].

It is currently included in the diagnostic work-up of CIEDI with two main indications: (1) the confirmation of the presence of the infection in the early stages of the diagnostic framework, when other imaging techniques do not allow for a definitive diagnosis; and (2) the detection of septic emboli because it guarantees a whole-body examination. In addition, ^18^F-FDG PET/CT can be considered an imaging method for evaluating response to therapy and monitoring complications; the comparison between consecutive tests allows to verify any reduction in the intensity of radioglucose uptake, indicative of a lower intensity of the phlogistic/infectious process, therefore disease resolution [23,24].

The inclusion of ^18^F-FDG PET/CT in CIEDI management has also demonstrated a substantial reduction in the rate of misdiagnosis of CIED-related infectious endocarditis (CIED-RIE) [17,18,25]. In particular, when cardiac involvement occurs, CIED-RIE becomes a complex nosological entity, whose management requires the activation of a multidisciplinary group of expert medical figures, called the Endocarditis Team, who actively cooperate and collaborate together with their own specific experiences and skills, in order to guarantee the best management of patients suffering from CIED-related endocarditis [17,26,27].

The main aim of the present study was to determine the diagnostic performance and reliability of the qualitative analysis of ^18^F-FDG PET/CT in the early diagnosis of CIEDI.

Furthermore, two secondary endpoints were evaluated: the usefulness of the semi-quantitative analysis of ^18^F-FDG PET/CT (which is still debated and not always recommended [28,29,30]), through the collection of various semi-quantitative parameters and the comparison of the variability of the relative ranges of values; and the validity of adequate dietary preparation of patients preparatory to the suppression of cardiac glucose metabolism, in order to guarantee optimal image quality for diagnostic purposes.

## 2. Materials and Methods

### 2.1. Patient Selection and Eligibility Criteria

In this retrospective observational study, 48 patients, who underwent ^18^F-FDG PET/CT for the clinical suspicion of CIED infection, were included.

All ^18^F-FDG PET/CT examinations were carried out in the period of time between December 2018 and December 2022. In all patients studied, the implantation of the device took place at least 3 months before the instrumental evaluation by ^18^F-FDG PET/CT, according to the recommended indications, to exclude any findings attributable to inflammation post-implantation/revision of the device [23].

All patients were clinically evaluated before carrying out the examination, using both laboratory and first level instrumental tests, and a suspicion of CIED infection was confirmed, without being able to exclude possible cardiac involvement.

CIEDI was suspected based on the presence of at least two of the following signs:

Clinical signs: fever > 38 °C; local signs of infection of the generator pocket (erythema and/or localized cellulitis and/or swelling and/or secretion and/or dehiscence and/or pain at the level of the pocket and/or collection of liquids and/or CIED exposure);

Laboratory signs: elevated values of inflammation indices: erythrocyte sedimentation rate (ESR) and/or C-reactive protein (CRP) and/or procalcitonin (PCT) and/or white blood cells (WBC) and/or presepsin; positivity to blood culture;

Instrumental signs: positive trans-thoracic echocardiography (TTE) and/or positive trans-esophageal echocardiography (TEE).

A large-spectrum antibiotic therapy was empirically established for all patients, according to current guidelines [17]; the execution of the ^18^F-FDG PET/CT exam was guaranteed within the first five days of starting therapy.

Informed consent for the collection and use of anonymous data for research purposes were obtained in written form from each patient at the time of first hospitalization, so no further ethics committee approval were required for the review of patient files and data.

### 2.2. ^18^F-FDG PET/CT

The acquisition of ^18^F-FDG PET/CT exams was performed following the reference guidelines of the European Association of Nuclear Medicine (EANM) and the European Association of Cardiovascular Imaging (AECVI) and the protocols recommended therein [31,32].

Standardized protocols have been prepared and applied in order to ensure the reproducibility of the examination. These standardized protocols have been adopted regarding the following aspects, explained below:

- dietary preparation of patients;
- imaging technique;
- image analysis.

#### 2.2.1. Dietary Preparation of Patients

All patients followed a specific dietary protocol in the 24–48 h prior to the ^18^F-FDG PET/CT execution, characterized by a high intake of fatty foods and a low intake of carbohydrates (High-Fat Low-Carbohydrate Diet, HFLC Diet). This ketogenic-like diet was set and specifically requested to obtain myocardial suppression of glucose metabolism and better visualization of the organ itself and the surrounding structures.

**Figure 1:**
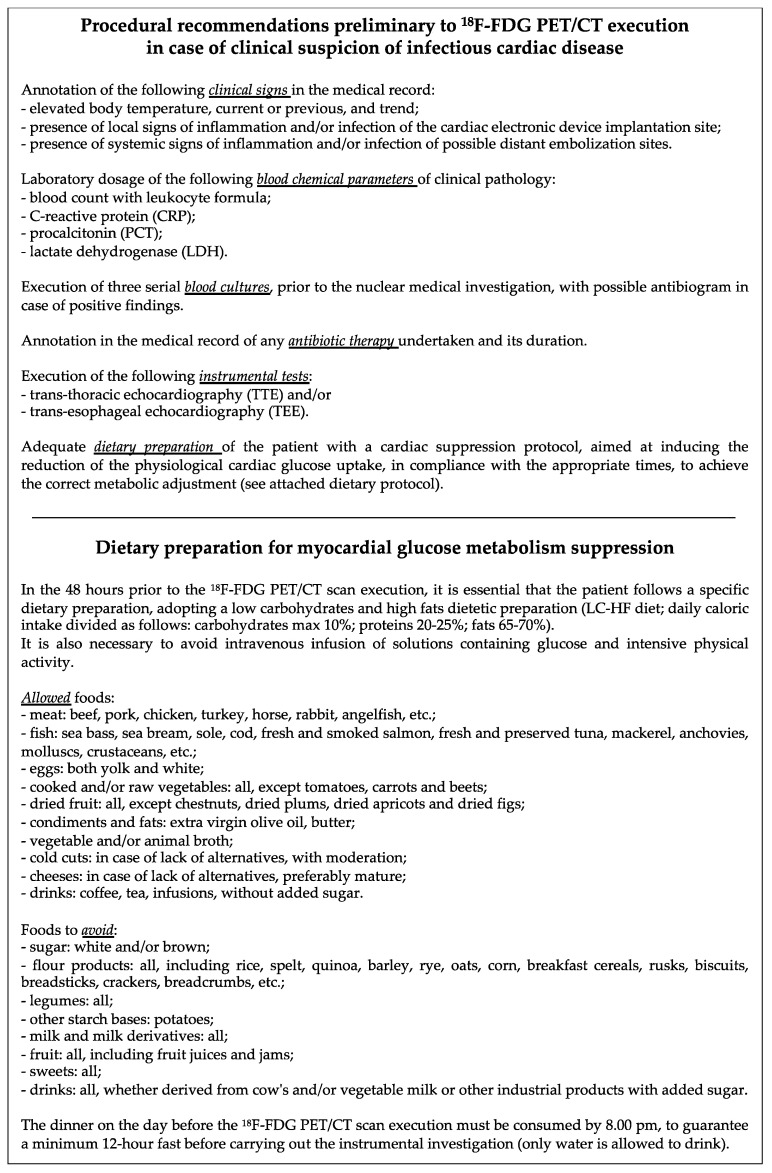
Procedural recommendations and dietary preparation for myocardial glucose metabolism suppression preliminary to ^18^F-FDG PET/CT execution.

In order to reach this specific purpose, a specific document was drawn up with all the information necessary for patients to be adequately prepared; these indications were made known to all the hospitalization departments of the patients included in this study cohort, where the medical-nursing team ensured their adequate implementation. The model of the document containing the procedural recommendations to be followed for the dietary preparation of patients is illustrated in Figure 1.

#### 2.2.2. Imaging Technique

On the day of ^18^F-FDG PET/CT, each patient was required to fast for at least 8 h before the exam; diabetes was not a reason for exclusion from this study sample, if under pharmacological control. After verifying that the blood glucose levels were lower than 140 mg/dL, the intravenous administration of radioglucose was carried out, with a dose of 2.5 MBq/kg, in compliance with the EANM guidelines [31].

After 45–60 min, during which the patients guaranteed a minimum level of hydration (500 mL of water), the images were acquired using a combined multimodal Discovery LSA PET/CT scanner (GE Healthcare, Waukeska, WI, USA), which integrates a PET with a 16-slice low-dose CT scanner, necessary to perform attenuation correction and anatomical reconstruction of PET images.

PET images were obtained whole-body in a cranio-caudal direction, from the external acoustic meatus to the root of the thigh; PET scans were reconstructed with a 128 × 128 matrix with a maximum iterative reconstruction algorithm with ordered subsets (two iterations, 28 subsets), an 8 mm Gaussian filter and a 50 cm field of view.

The CT acquisition parameters were as follows: section thickness 3.75 mm; 350 mA; 120 kV; tube rotation time 0.8 ms; collimation field of view (FOV) 50 cm. The CT images were reconstructed with a filtered-back projection. Patients were not administered any iodinated intravenous contrast.

#### 2.2.3. Image Qualitative and Semi-Quantitative Analysis

Images from all ^18^F-FDG PET/CT examinations were blindly reviewed by two nuclear physicians with more than five years of experience, using the MultiVol PET/CT program (Volume Share 4.7 with Volume Viewer Software) installed on an Advantage Workstation (GE Healthcare, Waukesha, WI, USA). The ^18^F-FDG PET/CT images were displayed in three orthogonal planes as PET images, CT images, and fusion images. Image analysis was evaluated both with and without attenuation correction; the nonattenuated images were those used for the final qualitative interpretation, in order to avoid artifacts induced by metallic components of CIED.

Qualitative visual analysis was performed to define whether ^18^F-FDG PET/CT was positive or negative for CIED infection; this preliminary evaluation was carried out mainly on the three-dimensional reconstruction of maximum intensity projection (MIP). Both intensity and pattern distribution of glucose uptake were considered important factors to define the presence of altered tracer distribution. A test characterized by a nonhomogeneous and intense glucose uptake around the device (generator pocket and/or electrocatheters) greater than the activity of the mediastinal blood pool, was defined as positive (CIEDI+). A test was defined as negative in case ^18^F-FDG increased uptake was detected around the device, when compared to the activity of the surrounding tissues or the mediastinal blood pool (CIEDI−) [33,34,35]. In cases of discordant interpretation, the analysis was discussed and solved by reaching a shared consensus.

As regards semi-quantitative analysis, in the present study it was considered useful to carry out a semi-quantitative evaluation by collecting the following parameters: maximum standardized uptake values (SUV_max_), semi-quantitative ratio (SQR), and target-to-background ratio (TBR).

The semi-quantitative parameters were obtained from volumes of interest (VOIs) drawn semi-automatically near the regions under study; image reconstruction was also performed in the sagittal and coronal planes to ensure correct positioning of the VOIs.

The SUV_max_, normalized on the basis of the administered dose and the patient’s weight, was collected on the VOIs with the highest value drawn in correspondence with the generator pocket and along the path of the leads.

Two ratios of semi-quantitative values were subsequently calculated to obtain a background value for ^18^F-FDG absorption and evaluate the normalization of SUV_max_. The SUV_max_ was therefore measured in correspondence with two areas without significant activity and compared to the SUV_max_ obtained in the proximity of the CIED and/or the leads.

The SQR was defined as the ratio between the SUV_max_ obtained in the region surrounding the CIED and the average of the SUV_max_ of the normal right and left lung parenchyma; pathological lung parenchyma was excluded from in the analysis.

The TBR was calculated as the ratio between the SUV_max_ obtained in the region surrounding the CIED and the SUV_max_ of the lumen of the ascending aorta; areas of altered vascular wall or including periluminal prosthetic material were excluded in the analysis.

Areas of extra-cardiac uptake of the radiopharmaceutical were searched as possible locations suggestive of systemic septic embolization. The general clinical conditions of the patients and their clinical history were taken into consideration during the evaluation and interpretation of the areas of increased extra-cardiac glucose uptake.

A further qualitative evaluation was subsequently carried out in order to evaluate the patients’ compliance with the dietary preparation. The images of each exam were reviewed, and a qualitative judgment was attributed to the degree of myocardial suppression, according to a scale of three judgments: excellent, good, and poor, depending on whether the intensity of myocardial glucose uptake was respectively lower, equal to or greater than the glucose uptake detectable in the liver [36]. Cases in which the preparation was so poor to be non-diagnostic, due to widespread radiopharmaceutical uptake throughout the myocardium, were not included in the analysis.

### 2.3. Evaluation of Patients’ Outcome and Definition of the Gold-Standard Reference Method

The therapeutic choice was decided by a medical-surgical team; therapeutic decisions were made in all cases by the same medical specialist and established on the basis of current clinical guidelines [9,17,25,37].

In patients undergoing surgery to remove the device, the final diagnosis was made on the basis of the microbiological analysis of the removed material; in patients for whom surgical treatment was not performed, the final diagnosis was established through clinical-instrumental follow-up, according to the modified Duke’s criteria (mDC) [38,39]. Therefore, the gold-standard reference methods were respectively the result of the microbiological examination and the clinical decision of the medical team.

All patients were followed for at least six months after the ^18^F-FDG PET/CT, both in the case of surgical removal of the CIED and in the case of clinical monitoring only.

### 2.4. Statistical Analysis

The following diagnostic performance parameters of ^18^F-FDG PET/CT were calculated: sensitivity (Se), specificity (Sp), positive predictive value (PPV), negative predictive value (NPV), and diagnostic accuracy (DA).

The reproducibility of qualitative image analysis between observers was assessed with Cohen’s κ test.

The semi-quantitative parameters were evaluated using the Student *T*-test, to establish whether there was a statistically significant difference for each parameter in the two groups of patients identified by the visual analysis of the ^18^F-FDG PET/CT instrumental examination (CIEDI+, CIEDI−).

The ROC curves were obtained in order to estimate the cut-off values of the semi-quantitative parameters calculated, potentially allowing them to predict CIED infection.

Finally, curves were developed to evaluate the trend of image quality over time with regards to dietary preparation aimed at myocardial suppression and to assess whether there had been an improvement in patients’ compliance with the recommendations provided.

All statistical evaluations were performed using SPSS^®^ Software, version 25.0 (SPSS, Chicago, IL, USA).

## 3. Results

### 3.1. Characteristics of this Study Sample

The main characteristics of the patients belonging to this study sample are shown in Table 1.

The average time between CIED implantation and ^18^F-FDG PET/CT was 3.2 years (range: 6 months–7 years).

The hospitalization departments from which requests for evaluation were received were the following: hospital cardiology (21/48; 43.7%); university cardiology (13/48; 27.1%); university nephrology (4/48; 8.3%); internal medicine (3/48; 6.2%); university endocrinology (2/48; 4.2%); cardiac surgery (2/48; 4.2%); pulmonology (1/48; 2.1%); infectious diseases (1/48; 2.1%); and university orthopedics (1/48; 2.1%).

Clinically, 24/48 (50%) patients had fever upon hospital admission and 18/48 (37%) showed local signs of infection of the generator pocket.

Laboratory tests showed WBC values were outside the reference range in 8/48 (16.7%) patients, ESR values in 13/24 (54.2%) patients, CRP values in 36/45 (80%) patients and those of PCT in 23/42 (54.8%) patients. Presepsin levels were required in 15 patients and were increased in almost all subjects (13/15; 86.7%).

**Figure 2:**
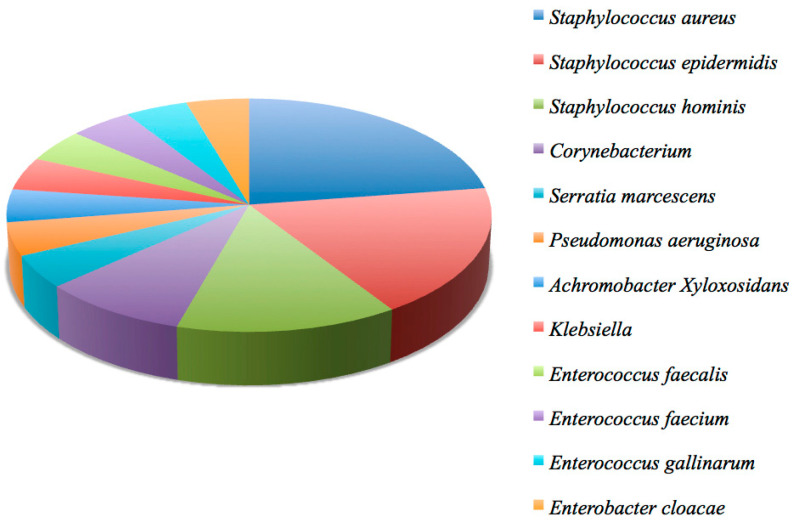
A graphic representation of the microbiological analysis results from blood cultures.

Blood cultures were requested and performed for all patients; they were positive in 18/48 (37.5%) patients and negative in the remaining 30/48 (62.5%). The bacteria isolated from the cultures, responsible for the bacteremia, were: *Staphylococcus aureus* in 5/18 (27.8%); *Staphylococcus epidermidis* in 4/18 (22.2%); *Staphylococcus hominis* in 3/18 (16.7%); *Corynebacterium* in 2/18 (11.1%); *Serratia marcescens* in 1/18 (5.6%); *Pseudomonas aeruginosa* in 1/18 (5.6%); *Achromobacter Xyloxosidans* in 1/18 (5.6%); *Klebsiella* in 1/18 (5.6%); *Enterococcus faecalis* in 1/18 (5.6%); *Enterococcus faecium* in 1/18 (5.6%); *Enterococcus gallinarum* in 1/18 (5.6%); *Enterobacter cloacae* in 1/18 (5.6%) (Figure 2) [2]. In 4/18 (22.2%) patients the bacteremia was found to be supported by two species simultaneously.

The instrumental tests performed before the ^18^F-FDG PET/CT were transthoracic echocardiography (TTE) and, where possible based on the clinical conditions of the patients, trans-esophageal echocardiography (TEE). TTE, performed in 33/48 (68.7%) patients, was positive in 12/33 (36.4%) cases, while TEE, performed in 22/48 (45.8%) patients, was positive in 19/22 (86.4%) cases.

The average time between the diagnosis of suspected CIED infection and the performance of the ^18^F-FDG PET/CT examination was 2 days (range: 1–4 days).

During this time, empiric antibiotic therapy was established for patients, according to guidelines; the most common used antibiotics were the following, used both individually and in combination: cefazolin, amoxicillin + clavulanic acid, levoxacin, daptomycin, teicoplanin + ceftriaxone, piperacillin tazobactam, meropenem + linezolid and azithromycin. The antibiotic therapy did not cause interference with the results of the studies on the microbiological samples and did not invalidate the results of the ^18^F-FDG PET/CT, as the time interval between the start of the medical therapy and the execution of the instrumental examination was not sufficient to obtain a complete reduction in the bacterial count responsible for any interference with glycidic uptake.

All patients underwent the myocardial suppression dietary protocol in the 24–48 h before the examination.

### 3.2. Results of the ^18^F-FDG PET/CT Examination Analysis

**Figure 3:**
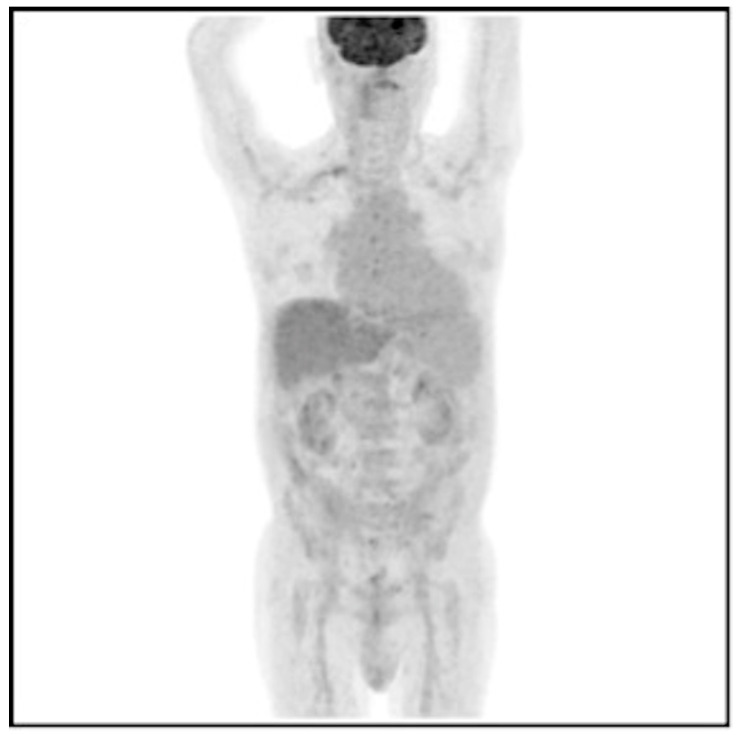
^18^F-FDG PET/CT of a 64-year-old man with implanted biventricular ICD, with suspected CIEDI due to alteration of inflammation indices, despite negative blood culture. The MIP projection shows the absence of areas of increased ^18^F-FDG uptake attributable to infection both in correspondence with the pocket and along the course of the leads.

The visual qualitative analysis of the acquisitions allowed us to define 29/48 (60.4%) ^18^F-FDG PET/CT exams as positive (CIEDI+) and the remaining 19/48 (39.6%) exams as negative (CIEDI−) (Figure 3).

**Figure 4:**
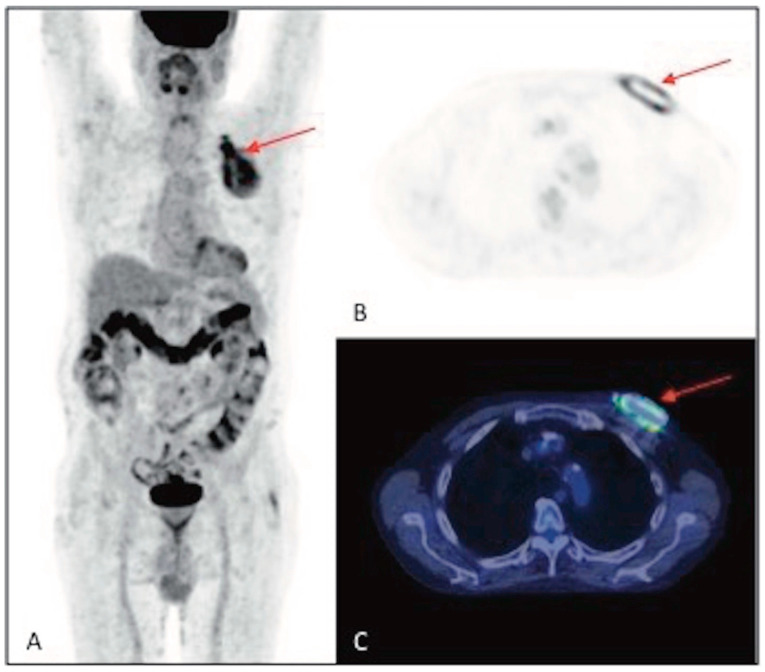
^18^F-FDG PET/CT of a 73-year-old man with implanted ICD, with suspected CIEDI due to the presence of local signs of infection and positive blood culture for *Staphylococcus aureus*. (**A**) MIP, (**B**) axial PET and (**C**) axial fusion images demonstrate the presence of intense and nonhomogeneous increased ^18^F-FDG uptake around the device, at the generator pocket (SUV_max_ 12.9) (red arrows). After the extraction of the device, microbiological analysis confirmed the infection caused by the pathogen *Staphylococcus aureus*.

**Figure 5:**
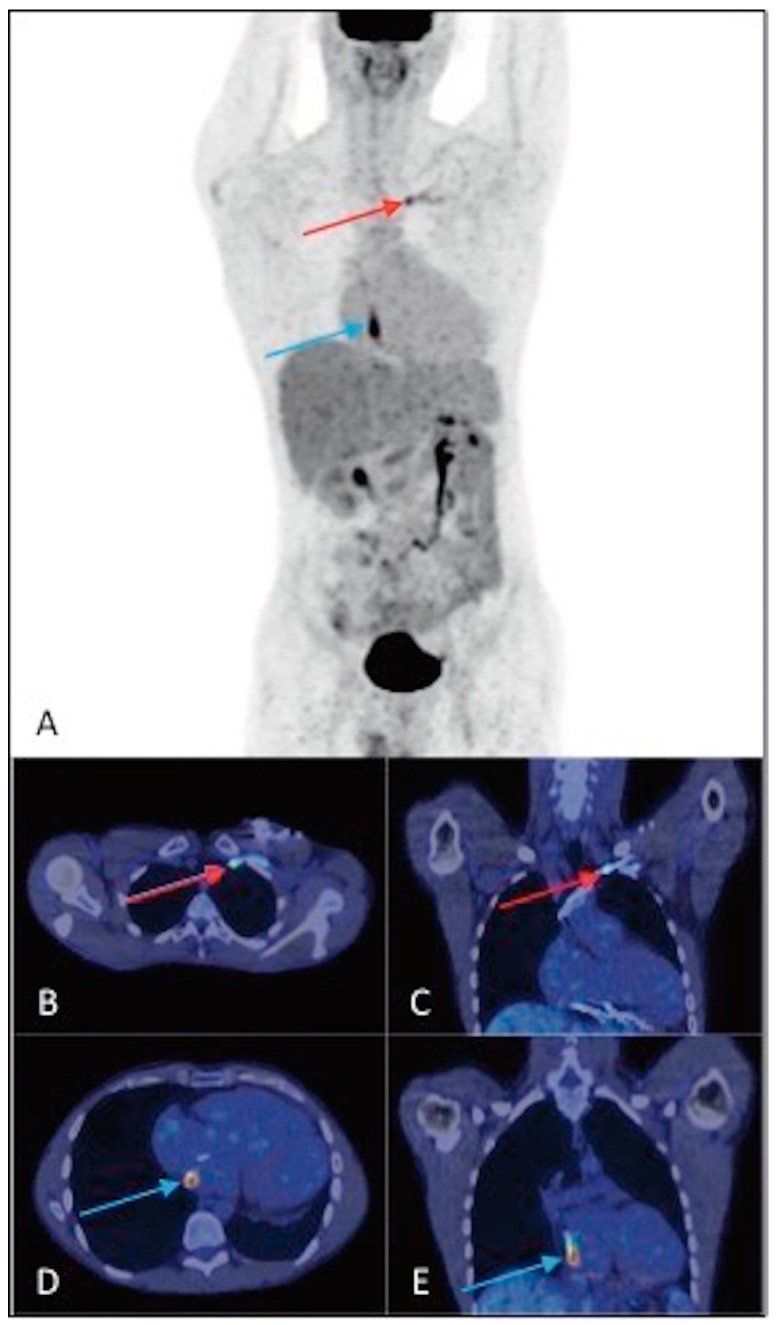
^18^F-FDG PET/CT of a 35-year-old man with implanted ICD, with suspected CIEDI due to persistent alteration of inflammation indices and positive blood culture for *Staphylococcus epidermidis*, in the absence of fever and local signs of infection. The (**A**) MIP, (**B**,**D**) axial fusion and (**C**,**E**) coronal fusion images demonstrate the presence of areas of intense and focal increased ^18^F-FDG uptake along the lead pathway, both at the retroclavicular level (SUV_max_ 6.4) (red arrows) and at the intrathoracic level (SUV_max_ 15.6) (blue arrows). The microbiological analysis, following the extraction of the device, confirmed the infection sustained by the pathogen *Staphylococcus epidermidis*.

**Figure 6:**
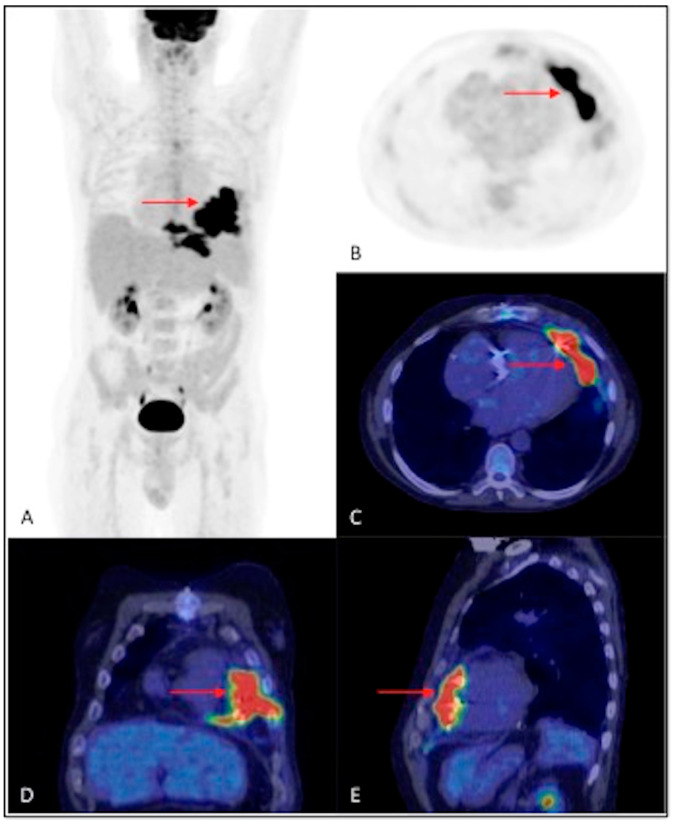
^18^F-FDG PET/CT of a 48-year-old man with implanted PM, with suspected CIEDI for therapy-resistant hyperpyrexia and positive blood culture for *Pseudomonas aeruginosa*. (**A**) MIP, (**B**) axial PET, (**C**) axial fusion, (**D**) coronal fusion, and (**E**) sagittal fusion images demonstrate the presence of intense hyperaccumulation of ^18^F-FDG along the pericardial part of the lead (SUV_max_ 25.5) (red arrows). The patient underwent extraction of the device and microbiological analysis confirmed the bacterial infection.

The sites of infection were found to be the generator pocket alone in 17/29 (58.6%) cases, the extracardiac course of the leads alone in 8/29 (27.6%), and both the pocket and a section of the lead leads in the remaining 4/29 (13.8%) (Figure 4, Figure 5 and Figure 6).

The semi-quantitative analysis allowed for the estimation of the SUV_max_ values at the site of suspected infection and the SQR and TBR ratios.

From the visual analysis of the ^18^F-FDG PET/CT exams, no areas of increased glucose uptake were detected in any extracardiac site, so cases of embolic localizations of distant disease were excluded.

**Figure 7:**
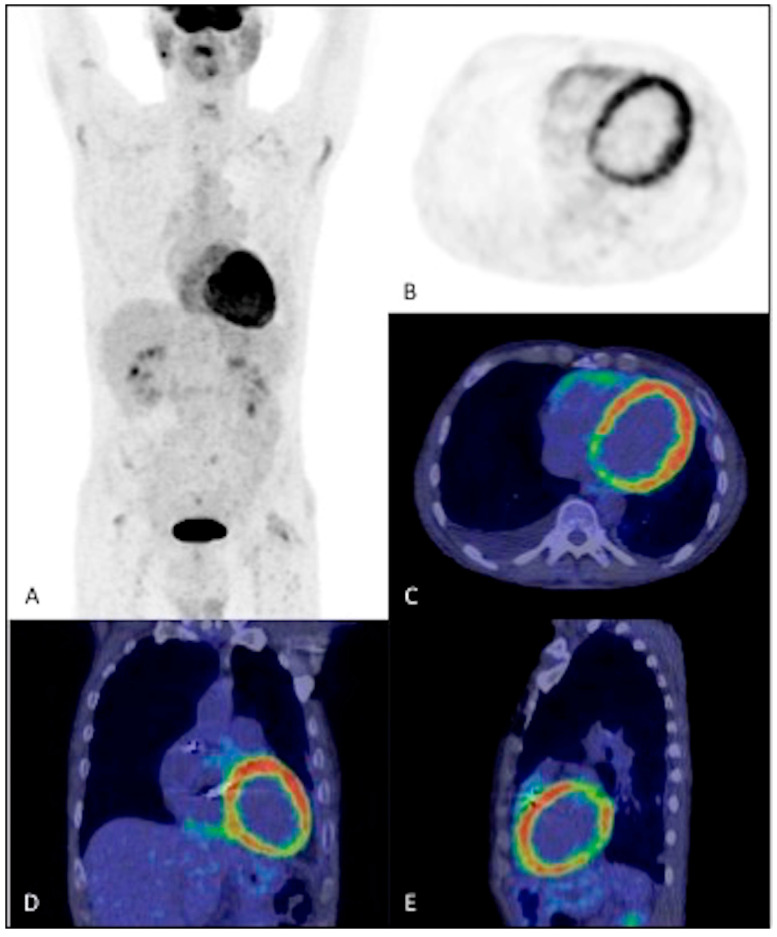
^18^F-FDG PET/CT of a 66-year-old man with a dual-chamber ICD with suspected CIEDI. (**A**) MIP, (**B**) axial PET, (**C**) axial fusion, (**D**) coronal fusion, and (**E**) sagittal fusion images demonstrate widespread glucose uptake throughout the myocardium, indicative of poor adherence to the dietary protocol for myocardial suppression; these images are considered of poor quality. The ^18^F-FDG PET/CT was considered negative for CIEDI but it was revealed to be a false negative.

The qualitative judgment attributed to the degree of myocardial suppression, according to a scale of three judgments, was found to be: excellent in 22/48 (46%) patients, good in 16/48 (33%), and poor in the remaining 10/48 (21%) (Figure 7 shows an example of poor quality images).

### 3.3. Patients’ Outcome and Diagnosis Using the Gold Standard

The cardiac device was explanted in 30/48 (62.5%) patients; the entire electrostimulation system was removed intravenously.

For these patients undergoing CIED’s surgical removal, the operation was planned promptly and carried out in an average period of 5 days (range: 3–7 days) following the performance of the ^18^F-FDG PET/CT.

The removed material was examined, and the microbiological analysis using bacterial culture allowed a final diagnosis to be made, which confirmed or excluded the presence of infection. The analysis was positive in 23/30 (76.7%) patients and negative in the remaining 7/30 (23.3%).

**Figure 8:**
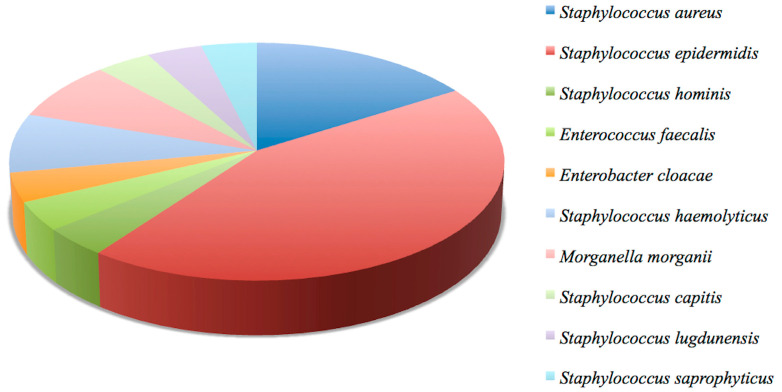
Graphic representation of the microbiological analysis results from the removed material.

The bacteria isolated were the following: *Staphylococcus epidermidis* in 11/23 (47.8%); *Staphylococcus aureus* in 4/23 (17.4%); *Staphylococcus haemolyticus* in 2/23 (8.7%); *Morganella morganii* in 2/23 (8.7%); *Staphylococcus capitis* in 1/23 (4.3%); *Staphylococcus hominis* in 1/23 (4.3%); *Staphylococcus lugdunensis* in 1/23 (4.3%); *Staphylococcus saprophyticus* in 1/23 (4.3%); *Enterococcus faecalis* in 1/23 (4.3%); *Enterobacter cloacae* in 1/23 (4.3%) (Figure 8). In 2/23 (8.7%) patients the infection was found to be supported by two species simultaneously.

For the remaining 18/48 (37.5%) patients who did not undergo surgery, clinical follow-up lasted 3 months; the re-evaluation took place according to the modified Duke’s criteria and the final clinical decision taken by the medical team of specialists confirmed negativity for infection in 15/18 (83.3%) patients.

### 3.4. Results of the Statistical Analysis

From the comparison of the results of the ^18^F-FDG PET/CT examination with the gold-standard reference method, 25/48 (52.1%) positive examinations were confirmed by the final diagnosis (True Positive, TP), while 4/48 (8.3%) were not substantiated (False Positive, FP). Regarding the negative ^18^F-FDG PET/CT exams, 18/48 (37.5%) were confirmed due to the absence of infection (True Negatives, TN), while only 1/48 (2.1%) was in contrast with the final diagnosis (False Negative, FN).

All the FP and FN ^18^F-FDG PET/CT exams were included in the poor quality image judgment.

The diagnostic performance of the ^18^F-FDG PET/CT exam was estimated by calculating the following parameters: Se 96.2% (95% CI: 80.36–99.90%); Sp 81.8% (95% CI: 59.72–94.81%); PPV 86.2% (95% CI: 71.97–93.83%); NPV 94.7% (95% CI: 72.28–99.20%); DA 89.6% (95% CI: 77.34–96.53%) (Table 2).

The reproducibility of the visual qualitative analysis between the two observers was excellent (K value = 0.89).

The mean values of the semi-quantitative parameters were calculated for the entire study population and the results are as follows: 4.33 for SUV_max_ (range: 0.93–25.5; SD: +4.21); 6.05 for the SQR (range: 1.26–32.28; SD: +5.96); 1.85 for the TBR (range: 0.47–11.33; SD: +1.86).

Furthermore, the mean values of the parameters described above were quantified in the two groups of patients identified based on the visual analysis of the ^18^F-FDG PET/CT examination. The values obtained were in CIEDI+ patients: 6.05 for SUV_max_ (range: 2.2–25.5; SD: +4.67); 8.48 for the SQR (range: 2.88–32.28; SD: +6.62); and 2.58 for TBR (range: 0.97–11.33; SD: +2.09). The values in CIEDI− patients were: 1.7 for SUV_max_ (range: 0.93–2.56; SD: +0.5); 2.34 for the SQR (range: 1.26–3.73; SD: +0.74); and 0.73 for TBR (range: 0.47–1.26; SD: +0.22).

The Student *T*-test showed a statistically significant difference (*p* < 0.05) for the mean values of each parameter: SUV_max_ (*p* = 0.014); SQR (*p* = 0.005); and TBR (*p* = 0.011).

**Figure 9:**
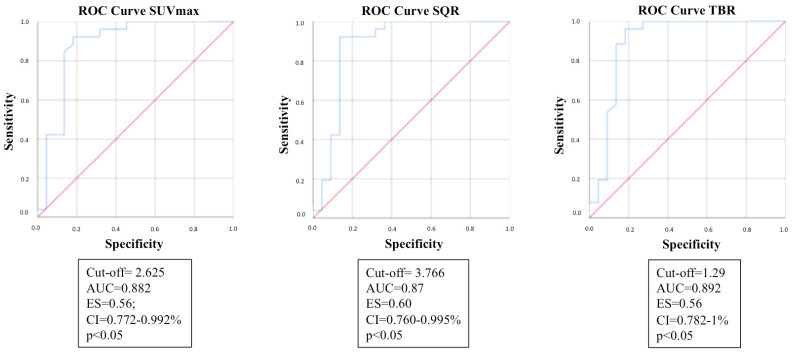
ROC curves of the following semi-quantitative parameters: SUV_max_; SQR; TBR.

Figure 9 reports the results of semi-quantitative parameters.

**Figure 10:**
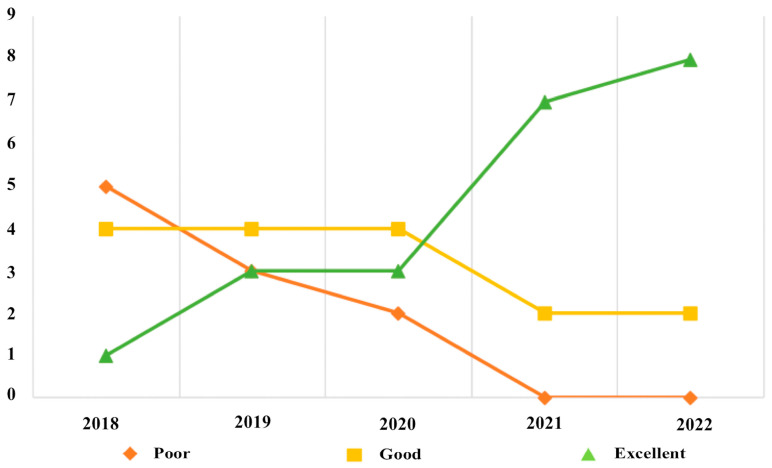
A graphic representation of the trend over time of the degree of adherence to the myocardial suppression dietary protocol.

The analysis of the trend over time of the degree of adherence to the myocardial suppression dietary protocol made it possible to obtain curves based on the number of examinations carried out per year and the degree of adherence expressed through evaluation of the global quality of the images (Figure 10).

## 4. Discussion

^18^F-FDG PET/CT, universally validated in the evaluation and management of numerous neoplastic pathologies, has also assumed a prominent position in this study of patients suffering from inflammatory and infectious diseases [20].

Suspected CIEDI is a clinical condition generally studied with traditional imaging techniques, among which echocardiography plays a pivotal role. However, a definitive diagnosis is not easy, especially in the short time necessary to establish a targeted and personalized therapy [40,41].

The aim of the present study was to validate the role of ^18^F-FDG PET/CT in the management of patients with suspected CIEDI, studying the results of both qualitative and semi-quantitative analysis. The fundamental aspect was standardization: each phase of this study was conducted respecting pre-established criteria, to normalize this study methodology as much as possible and to encourage uniformity in the interpretation of the results and reproducibility over time.

In the literature, the performance values of ^18^F-FDG PET/CT in the evaluation of CIEDI are quite variable, with different values depending on whether infection of the device pocket alone or infection of the electrocatheters and CIED-related endocarditis are considered [5,18,24,42].

In a recent large meta-analysis conducted by Mahmood et al., which involved 14 studies with a total of 492 patients, the sensitivity and specificity of ^18^F-FDG PET/CT in the diagnosis of CIEDI were 85% and 90% respectively, with higher values for the diagnosis of CIED pocket infections (Se: 96%; Sp: 97%) than those for the diagnosis of leads or CIED-related infectious endocarditis (Se: 76%; Sp: 83%) [43].

A second meta-analysis conducted by Juneau et al. confirmed the high diagnostic performance of ^18^F-FDG PET/CT for the diagnosis of CIED infections (Se: 87%; Sp: 94%), confirming higher values for the diagnosis of pocket infection (Se: 93%; Sp: 98%) compared to those for the diagnosis of lead infection and CIED-related endocarditis (Se: 65%; Sp: 88%) [44].

In our work, the sensitivity result (96.2%) is better than those reported in the literature, while specificity, although high (81.8%), is lower, as is the diagnostic accuracy (89.6%).

As regards the diagnosis of extracardiac complications, generally linked to embolization of endocarditic vegetations, the sensitivity was variable, while the specificity was 80% [45].

False negatives are possible and could be related to the presence of conditions that alter glucose metabolism and/or drugs that modify glucose sensitivity. It has been hypothesized that the reduction in sensitivity may be due to:

1. presence of small-sized vegetation along the pathway of the electrocatheters, below the spatial resolution power of the imaging technique;
2. Start of antibiotic therapy before carrying out the instrumental investigation [5,16,24,46].
3. Diffuse uptake of radioglucose by myocardial tissue;

Small-sized vegetation detection with PET is a challenge, however, the coregistration with the CT images, with a thickness of 3.75 mm, guaranteed the best spatial resolution obtainable, combining the metabolic data with an anatomical localization useful for the purposes of image interpretation. Furthermore, the use of late acquisitions, has been proposed to ensure a reduction in background blood activity and an increase in contrast with the background with increased sensitivity from 51% to 70%, without affecting the specificity [47]. This expedient, however, has not been validated in the guidelines, so its execution is not routine in standard image acquisition protocols.

With regard to the antibiotic therapy it can be a biased but literature reports that it does not interfere with the results of the 18F-FDG PET/CT examinations if undertaken in a short period of time, since in a few days of therapy, the local infection is not yet solved. In our study the mean time of antibiotic therapy was 2 days (range 1–4), and it was not sufficient to obtain a complete reduction in the bacterial count responsible for any interference with glycidic uptake. Furthermore this period of time is lower than the 7 days reported a possible cut-off for antibiotic interference in 18F-FDG uptake [5,48].

^18^F-FDG PET/CT shows greater diagnostic performance in patients who perform adequate dietary preparation for the suppression of the physiological absorption of radioglucose by the myocardium. Good compliance with dietary recommendations by patients allows for obtaining good image quality, so that there is no risk of interference attributable to physiological glucose uptake [5,42]. Otherwise in our study, the unique FN was due to poor quality images.

In order to overcome the limitations of cardiac ^18^F-FDG physiologic uptake, a protocol for myocardial suppression of glucose uptake was applied. The document illustrated in Figure 1 was provided to each hospital department requiring ^18^F-FDG PET/CT at least 24 h in advance.

Otherwise the finding of false positives can be attributed to the high ^18^F-FDG uptake linked to the presence of an aseptic tissue inflammatory reaction [35], as well as what happened in our study.

In our study, the reliability of the qualitative visual analysis of the ^18^F-FDG PET/CT images performed by the two nuclear medicine physicians was excellent (K = 0.89). The high quality of the images, both corrected and uncorrected for attenuation, avoided artifacts related to the metal components of the device [49]. Our results confirmed those of Granados et al. and Bensimhon et al., in whose studies, the K values for the presence of CIED infection were 0.81 and 0.8, respectively [33,49].

Literature reports extreme heterogeneity in semi-quantitative analysis of the ^18^F-FDG PET/CT both for the parameters analyzed and in the results observed: some studies investigated SUV_max_, while others proposed the use of ratios of values between a target region and the background [40]. In our study we analyzed both SUV_max_ and the ratio parameters SQR and TBR.

In most studies, SUV_max_ did not appear to be a useful parameter for discriminating the presence or absence of infection along the lead pathway and did not add any added value to the qualitative analysis [30,33,48,50]. Bensimhon et al. hypothesized that it was linked to patient-related factors that can influence glucose uptake. Differently, other studies reported both the SUV_max_ and the parameters obtained from the ratio with the background as significant in modifying the diagnostic performance of ^18^F-FDG PET/CT [34,47]. Bensimhon et al. proposed cut-off values to discriminate positive patients for infection from negative ones, they identified 2.56 for the SUV_max_ and 4.15 for the SQR as cut-off values [48].

In our study, the statistical evaluation of the semi-quantitative parameters was significant: all the three parameters investigated (SUV_max_, SQR and TBR) demonstrated a statistically significant difference when comparing the values of the group of patients with infection and those of patients without infection.

Furthermore, the cut-off values obtained from the ROC analysis of our study were also in line with those reported in the literature: 2.625 vs. 2.56 for the SUV_max_ and 3.766 vs. 4.15 for the SQR.

Considering the effect of an adequate protocol for myocardial suppression of glucose uptake, it should be stressed that the good results of our study are related to the high compliance of patients with the dietary preparation and procedural recommendations (Figure 1). From the beginning of this study to the end we observed a progressive increase in the number of excellent-quality exams, with an opposite trend regarding poor-quality exams, thanks to the introduction of the specific model disclosed to patients and clinicians. It is also demonstrated by the improved performance of the results in the present studies as compared to preliminary results previously published, when recommendations were provided orally and a specific model was not equipped [39].

It should be remembered that ^18^F-FDG PET/CT is a multimodal imaging method that explores the whole body; its most relevant advantages are the evaluation of the extracardiac components of the CIED, which are difficult to investigate by traditional echocardiography, the identification of unexpected sources of primary infection and the identification of distant embolic complications [34,51,52,53]. In our study, no sites of infection were detected in addition to those related to CIED components.

Although our work has shown promising results, it is not free from some limitations:

- the retrospective and monocentric nature of the analysis;
- small sample size, even if it was homogeneous and in line with the study samples reported in the literature.

## 5. Conclusions

Our results confirm the role of ^18^F-FDG-PET/CT in the management of patients suspected of CIEDI and its reliability in early diagnosis. The method has proven to be highly sensitive and specific, and has further advantages such as repeatability and non-invasiveness.

A medical-nursing team inclusive of clinicians and nuclear medicine experts allows the appropriate dietary preparation, which is fundamental for the improvement of image quality.

To date ^18^F-FDG-PET/CT is recommended in the management of patients with suspected CIEDI in the definition of the most appropriate diagnostic-therapeutic approach, especially in the early stages of evaluation of the pathology.

## Figures and Tables

**Table 1:** The main epidemiological characteristics of the patients included in the study sample.

Epidemiological Characteristics			
Sex	Men	38	
	Women	10	
Age	Average age	67 yrs	
	Age range	26–88 yrs	
Type of implanted device	PM	15/48	(31.2%)
	Dual-chamber PM	5/48	(10.4%)
	ICD	12/48	(25%)
	Dual-chamber ICD	11/48	(23%)
	Biventricular ICD	55/48	(10.4%)

**Table 2:** Diagnostic performance values of the 18F-FDG PET/CT exam.

Diagnostic Performance Values of the ^18^F-FDG PET/CT Exam		
Sensitivity (Se)	96.2%	(95% CI: 80.36–99.90%)
Specificity (Sp)	81.8%	(95% CI: 59.72–94.81%)
Positive Predictive Value (PPV)	86.2%	(95% CI: 71.97–93.83%)
Negative Predictive Value (NPV)	94.7%	(95% CI: 72.28–99.20%)
Diagnostic Accuracy (DA)	89.6%	(95% CI: 77.34–96.53%)

## Data Availability

Data are available after a request to the corresponding author.
